# Genetic diversity of the Central European wild boar (Sus scrofa scrofa) population and domestic pig (Sus scrofa domesticus) breeds based on a microsatellite DNA locus

**DOI:** 10.18699/VJ21.095

**Published:** 2021-12

**Authors:** E.A. Snegin, V.M. Makeeva, A.P. Kaledin, A.M. Ostapchuk, I.D. Alazneli, A.V. Smurov

**Affiliations:** Belgorod National Research University, Belgorod, Russia; Lomonosov Moscow State University, Moscow, Russia; Russian State Agrarian University – Moscow Timiryazev Agricultural Academy, Moscow, Russia; Russian State Agrarian University – Moscow Timiryazev Agricultural Academy, Moscow, Russia; Lomonosov Moscow State University, Moscow, Russia; Russian State Agrarian University – Moscow Timiryazev Agricultural Academy, Moscow, Russia

**Keywords:** wild boar, pig breeds, microsatellite loci, genetic structure, effective population size, кабан, породы свиней, микросателлитные локусы, генетическая структура, эффективная численность популяции

## Abstract

The results of studies of the genetic structure of the Central European wild boar (Sus scrofa scrofa) population and four breeds of domestic pigs (Duroc, Yorkshire, Large White and Landrace) bred in the Central Black Earth region of Russia are presented in this work. Based on 12 microsatellite loci, a significant ( p <0.05) decrease in the level of genetic variability in bred breeds was shown. The expected heterozygosity and Shannon index were as follows: in the wild boar, Ho = 0.763 ± 0.026, I = 1.717 ± 0.091; in the Duroc breed, Ho = 0.569 ± 0.068, I = 1.191 ± 0.157; in the Landrace, Ho = 0.618 ± 0.062, I = 1.201 ± 0.147; in the Large White, Ho = 0.680 ± 0.029, I = 1.362 ± 0.074; and in the Yorkshire, Ho = 0.642 ± 0.065, I = 1.287 ± 0.156. The results of checking genotypic Hardy–Weinberg equilibrium based on the G-test of maximum likelihood demonstrated that the overwhelming majority of loci in the wild boar population were in the state of said equilibrium. By contrast, in pig breed populations, some loci demonstrated a significant deviation from the indicated equilibrium. In addition, the Yorkshire, Large White, and Landrace populations had loci, for which the hypothesis of neutrality was reliably rejected based on the results of the Ewens–Watterson test. The revealed private alleles, characteristic of the wild boar and breeds, can later be used to identify them. The ordination of the centroids of different herds in the space of the first two principal coordinates based on the matrix of pairwise estimates of Nei’s genetic distances showed that the most distant populations are the Duroc and Boar breeds, and the most genetically close are the Yorkshire and Landrace breeds. The closest to the wild boar population was the Large White breed. The assessment of the effective size, carried out using the method based on the linkage disequilibrium and the molecular coancestry method, showed that in all studied groups, including the wild boar population, the effective size was less than 100 individuals. The low effective size of the wild boar population (Ne = 21.8, Neb = 4.0) is probably caused by the death and shooting of animals due to Pestis africana suum.

## Abstract (ru)

В работе приведены результаты исследований генетической структуры популяции централь- ноевропейского кабана (Sus scrofa scrofa) и четырех пород домашних свиней (дюрок, йоркшир, крупная бе- лая и ландрас), разводимых в Центрально-Черноземном регионе России. На основе 12 микросателлитных локусов установлено достоверное (р < 0.05) снижение генетической изменчивости в разводимых породах. Ожидаемая гетерозиготность и индекс Шеннона были равными: у кабана – Ho = 0.763 ± 0.026, I = 1.717 ± 0.091; у пород дюрок – Ho = 0.569 ± 0.068, I = 1.191 ± 0.157; ландрас – Ho = 0.618 ± 0.062, I = 1.201 ± 0.147; крупная бе- лая – Ho = 0.680 ± 0.029, I = 1.362 ± 0.074; йоркшир – Ho = 0.642 ± 0.065, I = 1.287 ± 0.156. Результаты проверки генотипического равновесия Харди–Вайнберга на основе G-теста максимального правдоподобия показали, что в популяции кабана большинство локусов находилось в состоянии генотипического равновесия Хар- ди–Вайнберга. Напротив, в популяциях различных пород свиней часть локусов демонстрирует достовер- ное отклонение от отмеченного равновесия. Кроме того, в популяциях йоркшир, крупная белая и ландрас присутствовали локусы, для которых достоверно отвергалась гипотеза о нейтральности на основании ре- зультатов теста Эвенса–Ваттерсона (Ewens–Watterson test). Обнаруженные приватные аллели, характерные для кабана и различных пород, в дальнейшем могут быть использованы для их идентификации. Ординация центроидов разных стад в пространстве первых двух главных координат на основании матрицы попарных оценок генетических дистанций М. Nei показала, что наиболее удаленные популяции – породы дюрок и кабан, а самые генетически близкие – йоркшир и ландрас. Ближе всех к популяции кабана была порода крупная белая. Оценка эффективной численности, проведенная с использованием метода, основанного на неравновесии по сцеплению (linkage disequilibrium) и MC-метода (the molecular coancestry method), проде- монстрировала, что во всех изученных группах, включая и популяцию кабана, эффективный размер оказался меньше 100 особей. Низкое значение эффективного размера популяции кабана (Ne = 21.8, Neb = 4.0), вероят- но, является следствием падежа и отстрела животных из-за африканской чумы свиней (Pestis africana suum).

## Introduction

According to various estimates, the domestication of the wild boar began 7–9 thousand years ago. During this time, more than 730 breeds of these animals have been created by man. It is obvious that for such a long “cultural” evolution, various breeds, being so-called clean lines, have largely lost the natural genetic potential that provides homeostatic mechanisms. As a result, maintaining the stability of existing breeds, like any artificially created systems, requires significant financial investments. In this regard, the study of the genetic potential of natural populations of wild boars for a possible increase in the resistance of pig breeds (for example, by methods of genome editing, etc.) is highly relevant.

In this regard, the study of wild boar genetics is now receiving much attention, both in Russia and other countries (Gladyr et al., 2009; Zinovieva et al., 2013; Rębała et al., 2016; Mihalik et al., 2020). In addition, due to constant outbreaks of the African swine fever (Pestis africana suum) in Russia, the wild boars are regularly shot as potential carriers of this disease. At the same time, the population structure of this animal is not taken into account, which can cause depletion of the gene pool and, against the background of increasing anthropogenic pressure, lead to the extinction of some groups. Examples of a significant reduction in the population of commercial species occurring due to the data on the state of the gene pool being neglected are well known (Altukhov, 2003).

Also, in the practice of molecular genetic laboratories, for forensic purposes, it is often necessary to diagnose tissue samples from illegally caught wild boars and prove their belonging to a wild species, and not to domesticated forms of pigs (Kipen et al., 2016; Lorenzini et al., 2020), or identify wild boar tissue in food (Szemethy et al., 2021). In this regard, the identification of private alleles for natural populations is also an urgent problem.

Microsatellite DNA loci (STR markers), which are tandem repeats of the noncoding part of nuclear DNA, are very convenient markers for studying genetic processes in populations. There are many works on the assessment of population gene pools of both domestic pigs and wild boars in different regions (Vernesi et al., 2003; Ferreira et al., 2009; Nikolov et al., 2009; da Silva et al., 2011; Choi et al., 2014; Sahoo et al., 2016; Ryabtseva et al., 2018; Han et al., 2021; Snegin et al., 2021).

The purpose of this work is to assess the genetic diversity of microsatellite loci in the population of the Central European wild boar (Sus scrofa scrofa) and the most common pig breeds (Sus scrofa domesticus) bred in the Central Black Earth region of Russia. It should be noted that such studies have not previously been conducted in this region.

## Materials and methods

A total of 320 animals were involved in the study. A sample of 30 wild boars was taken from the populations of the Oryol region (districts: Korsakovsky, Zalegoshchennsky, Novosilsky, Pokrovsky, Shablykinsky), Russia. The wild boars were caught during the hunting season of 2018. For comparison, samples from four populations of different breeds of domestic pigs bred on the farms of the Central Black Earth Region of Russia were used: Durok – 67 individuals (Belgorod region), Yorkshire – 108 individuals (Kursk region), Landrace – 50 individuals (Belgorod region), large white – 65 individuals (Voronezh region). All analyzed animals are Canadian breeds.

All 12 microsatellite loci recommended by ISAG-FAO (International Society for Animal Genetics, Food and Agriculture Organization) (FAO SoW-AnGR…, 2006) and arranged in one multiplex panel (S0101, S0155, S0228, S0355, S0386, SW24, SW240, SW72, SW857, SW911, SW936, SW951) were used as DNA markers (Table 1). Primers for PCR were selected taking into account the amplification of all 12 loci in one tube. The size of all amplified PCR products, taking into account all known alleles, was < 300 base pairs.

In the domestic pigs, DNA extraction was carried out from ear pits, and in the wild boars – from muscle tissue samples. For this purpose, we used kits with proteinase K DNA-Extran- 2 (Syntol, Russia). The PCR reaction was carried out on a Verity amplifier (Applied Biosystems, USA) in 20 μL of a mixture containing 20 ng of genomic DNA, PCR buffer (10 mmol Tris-HCl (pH 8.3), 50 mmol KCl, 2 mmol MgCl2), 0.25 mmol dNTP , 0.5 μmol primer, 1 unit Taq DNA polymerase (inhibited for hot start).

**Table 1.**
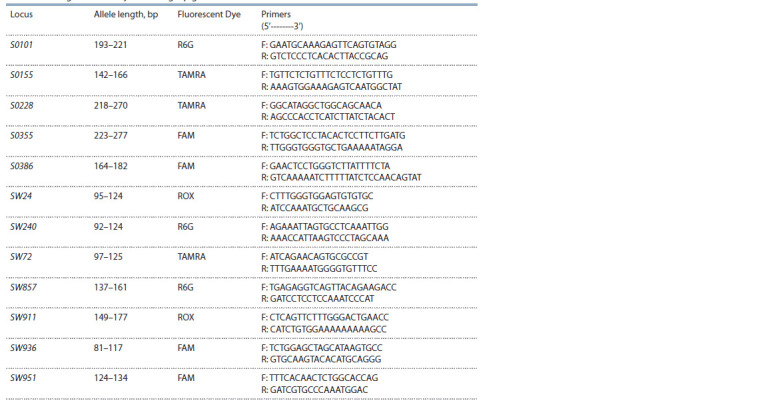
The characteristics of the microsatellite loci recommended by ISAG for determining the reliability of the origin pigs

PCR parameters: 94 °C – 3 min; (98 °C – 30 sec, 59 °C – 120 sec, 72 °C – 90 sec) – 4 cycles; (94 °C – 30 sec, 59 °C – 120 sec, 72 °C – 90 sec) – 6 cycles; (90 °C – 30 sec, 59 °C – 120 sec, 72 °C – 75 sec) – 20 cycles; 68 °C – 30 min. The heating rate from 59 to 72 °C was no more than 0.3 °C/1 sec.

Fragment analysis of the PCR products was carried out on an ABI PRISM 3500 automatic capillary DNA sequencer (Applied Biosystems, USA) using 50 cm capillaries and a POP-7™ polymer matrix. Fragment size analysis was performed using GeneMapper R Software v. 4.1 (Applied Biosystems).

For statistical processing of the data obtained we used the GenAIEx v. 6.5 (Peakall, Smouse, 2012) and PorGene 1.32 (Yeh et al., 1999).

## Results

In Table 2, the allele frequencies of the microsatellite loci used for analysis are presented. The data provide insight into the distribution of various alleles among populations of domestic pigs and wild boars.

**Table 2.**
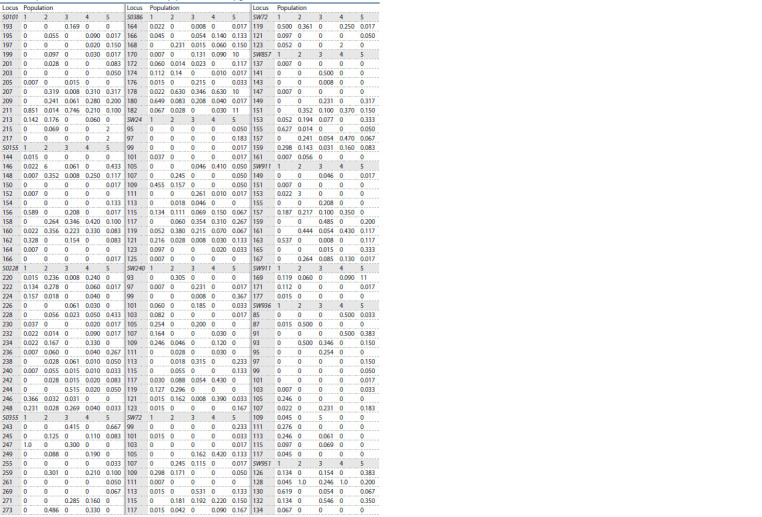
Frequencies of the microsatellite loci alleles in the populations of various pig breeds and wild boars Notе. Population: 1 – Duroc; 2 – Yorkshire; 3 – Large White; 4 – Landrace; 5 – Wild Boar.

The presence of private alleles in different populations is shown in Table 3. The results show a high content of unique alleles in the wild boar population (16 alleles). The highest frequency of private alleles was found at the loci SW24 and SW72 (0.25 in each). At the same time, the allele 97 at the SW24 locus, and the allele 99 at the SW72 locus are found more often than others among private alleles. The pig of the Duroc breed is slightly inferior to the wild boar in the number of private alleles, while the 105 and 111 alleles at the SW936 locus (0.246 and 0.276, respectively) had the highest frequency of occurrence. The Large White breed is almost three times inferior in the number of private alleles to the population of wild boar and Duroc pigs. However, some of the original alleles are found in this group of animals with noticeable frequency. For example, the allele 141, unique for this breed, at the SW857 locus was observed in half of the animals analyzed (frequency 0.5). No private alleles were found in the Landrace population, and only one private allele was noted in the Yorkshire population.

**Table 3.**
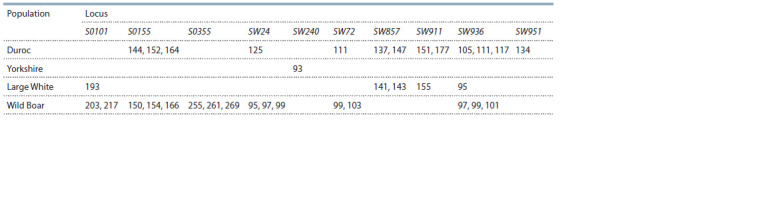
Private alleles in the studied populations of various breeds of pigs and wild boars Notе. No private alleles were found in the Landrace pig population.

The wild boar population has significantly high values of the indicators of genetic variability in comparison with the pig breeds. The comparison was carried out using the Pearson χ2 test ( p < 0.05) (Table 4).

**Table 4.**
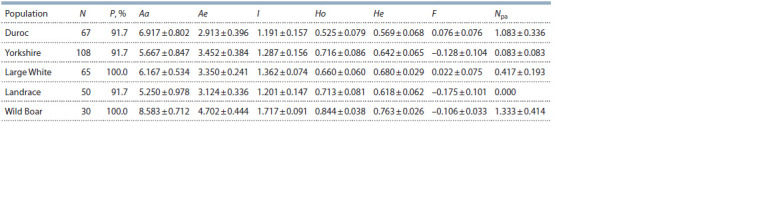
The indicators of the genetic diversity in the studied groups of pigs and wild boars (Mean ± SE) Notе. N – the number of individuals; P – the percentage of polymorphic loci; Aa – the average number of alleles; Ae – the effective number of alleles; I – Shannon’s index; Ho – observed heterozygosity; He – expected heterozygosity; F – the coefficient of inbreeding; Npa – the average number of private alleles per locus; Mean ± SE – mean ± standatd error.

The level of inbreeding (F ) in the studied groups turned out to be low, and the populations of Yorkshire, Landrace and wild boar received a negative value due to the prevalence of observed heterozygosity over the theoretically expected heterozygosity (see Table 4).

The results of checking the genotypic balance of Hardy– Weinberg based on the G-test of maximum likelihood demonstrated that in the wild boar population the overwhelming majority of the loci were in the state of the genotypic equilibrium of Hardy–Weinberg (Table 5). On the contrary, in the populations of the different breeds of pigs, some of the loci demonstrated a significant deviation from the indicated equilibrium. In addition, the monomorphic loci were noted in three groups (Duroc, Yorkshire, and Landrace). Also, in the populations of Yorkshire, Large White, and Landrace, the MC-loci were present, for which the hypothesis of neutrality was reliably rejected based on the results of the Ewens–Watterson test (Ewens, 1972; Watterson, 1978) (Table 6). Most of these loci were found in the Yorkshire and Landrace breeds.

**Table 5.**
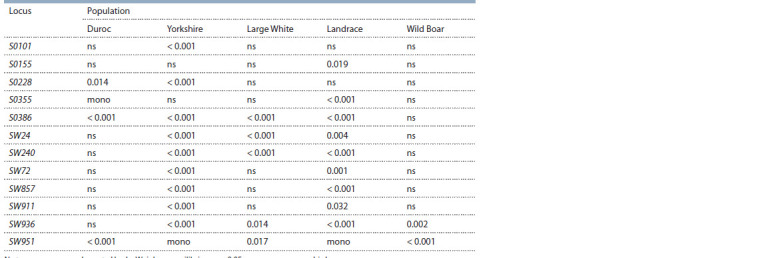
The results of checking the genotypic balance of Hardy–Weinberg for 12 loci of MS-DNA in herds of pigs of different breeds and wild boar based on the G-test of maximum likelihood (likelihood ratio G-test) Notе. ns – correspondence to Hardy–Weinberg equilibrium; p > 0.05; mono – monomorphic locus.

**Table 6.**
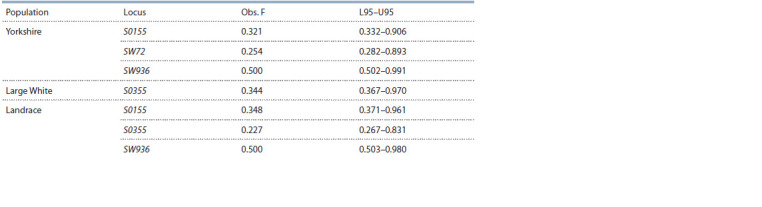
The results of the Ewens-Watterson test for 12 MS-DNA loci for various breeds of pigs and boars (only the loci for which the hypothesis of neutrality is reliably rejected are shown) Notе. Obs. F – the actual sum of squares of allele frequencies; L95, U95 – lower and upper values of the 95 % confidence interval of the Obs F estimate, calculated from 1000 simulations.

The degree of similarity of the genetic structure of the pigs was assessed using the principal component analysis, which resulted in the ordination of the centroids of different herds in the space of the first two Main Coordinates based on the matrix of pairwise estimates of genetic distances by M. Nei (Table 7, see the Figure). According to the data obtained, the most distant populations are the Duroc and Boar breeds, and the most genetically close are the Yorkshire and Landrace breeds. The closest to the wild boar population was the Large White breed.

**Table 7.**
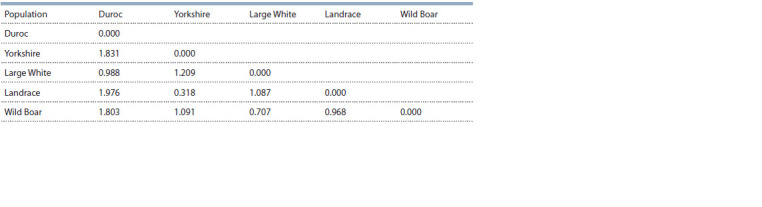
The values of genetic distances (according to М. Nei) between the studied groups of pigs and wild boars

**Fig.**
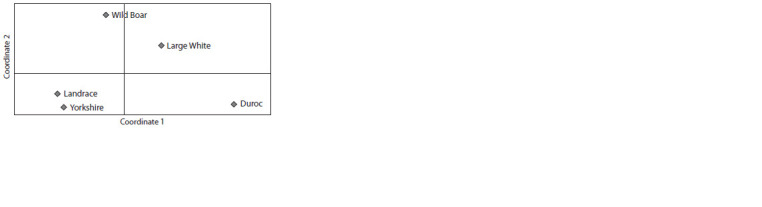
The ordination of centroids based on a matrix of pairwise estimates of M. Nei’s genetic distances based on the distribution of allele frequencies of 12 MS-DNA loci.

The effective population size was estimated using the linkage disequilibrium (LD) method (Hill 1981; Waples, 2006; Waples, Do, 2010), as well as the molecular coancestry method (Nomura, 2008). The calculations were performed using the NeEstimator V2 software (Do et al., 2014). The results are shown in Table 8.

**Table 8.**
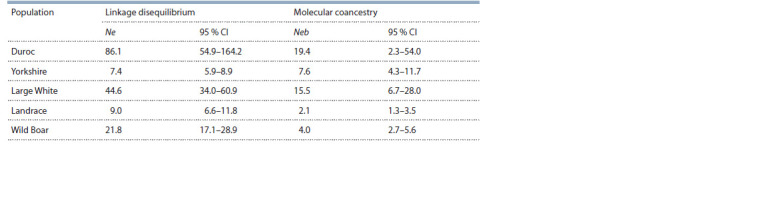
The estimates of the effective population size (Ne, Neb), calculated using the LD- and MC-method between 12 loci of MS-DNA Notе. 95 % CI – limits of 95 % conf idence interval.

## Discussion

The reliably high values of genetic variability ( p <0.05), noted in the wild boar population, are quite an expected phenomenon. This is despite the fact that the sample from the analyzed group was smaller than the samples from herds of domestic pigs. This clearly demonstrates the consequences of panmixis and genetic-automatic processes, which, in combination with the natural selection, form the gene pools of natural populations. Moreover, in the wild boar population we analyzed, the level of actual heterozygosity turned out to be significantly higher than in the populations of this animal both in Europe and Asia. At the same time, the Oryol group, in terms of the level of genetic diversity, turned out to be similar to the Chinese and Vietnamese wild boar populations (Table 9).

**Table 9.**
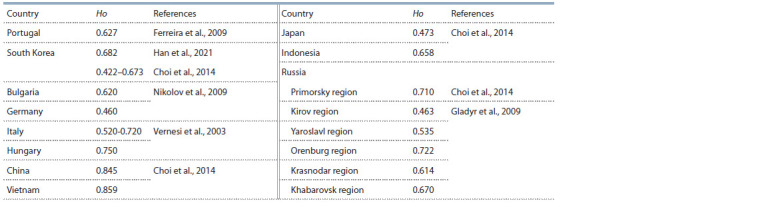
Level of actual heterozygosity for microsatellite markers in different wild boar populations

On the contrary, the transition of a number of loci to the monomorphic state and the lack of equilibrium according to Hardy–Weinberg, noted by us for most loci in domestic pig breeds, is a consequence of long-term artificial selection work, as a result of which many alleles of the “wild” type were lost, which led to the loss of genetic variety. This is also evidenced by the significant deviation from neutrality of some microsatellite loci in the Landrace, Yorkshire, and Large White breeds, which was revealed by us using the Evens–Watterson test (see Table 6).

Allelic diversity results in a large number of private alleles observed in the wild boar population. At the same time, the Duroc population, despite the large number of private alleles, turned out to be the most monomorphic among the studied groups. This probably indicates a long-term selection of this red color breed in the conditions of the North American continent, isolated from crossing with other pig breeds, including white breeds of European origin (Large White, Yorkshire and Landrace). This can explain the significant genetic distance of the Duroc breed, both from the wild boar and the European pig breeds. The presence of original alleles in the studied populations may be used in the future for identifying both the breed of pigs and belonging to a wild boar population.

It should be noted that the results obtained are in part consistent with the data obtained in the work of E.A. Glydyr et al. (2009). In this study, the level of actual heterozygosity in three out of five wild boar populations was found to be higher than in domestic pigs. However, the average number of the effective alleles was the same (Ae = 2.6). In terms of the number of private alleles, the wild boar population also surpassed the domestic pigs (21 versus 10, respectively).

The calculation of the effective population size based on the LD method showed that in almost all the studied groups, including the wild boar population, the effective size was less than 50 individuals. The only exception was the Duroc breed (Ne = 86.1). The data indicate the observed linkage disequilibrium, probably caused by closely related mating in the analyzed groups of domestic pigs. This genetic drift has generated a non-random association between alleles at different loci.

Calculations carried out using the MC method showed even lower values. This phenomenon is most likely associated with a small number of parental individuals (primarily males) who founded the studied populations. It is worth noting that a similar picture was obtained in the work on assessing the effective population size of the endangered Gochu Asturcelta pig breed (Menendez et al., 2016).

If the results for breeds of domestic pigs were comparable with data obtained in other studies, where Ne varied in general from 20 to 92 individuals (Šveistienė, Razmaitė, 2013; Krupa et al., 2015; Zanella et al., 2016; Lugovoy et al., 2018), then in relation to the wild boar population, the result was somewhat unexpected, since in previous studies Ne ranged from 180 to 1477 animals in natural populations (Cowled et al., 2008; Herrero-Medrano et al., 2013).

Such a low Ne value noted in the wild boar population of the Oryol region can be partly explained by a small sample, however, reasons behind it may be more serious. In particular, it is known that outbreaks of African swine fever (Pestis africana suum) (https://www.kommersant.ru/doc/4236233), resulting in the death of wild boars are often recorded on the territory of the Central Black Earth Region, which includes the specified area. In addition, in order to prevent the spread of infection, hunting farms are forced to shoot a significant part of the animals. It is likely that these phenomena affect the effective size of the wild boar populations.

## Conclusion

Thus, on the basis of the studies carried out, a clear reduction in the genetic diversity of the domestic pig breeds in comparison with the natural wild boar population was demonstrated. The presence of private alleles can further aid in the identification of wild boar and different breeds of pigs. Low values of the effective size of the studied groups require attention from breeders in relation to the breeds of pigs. In particular, the pig breeding companies in the region under study need to use a larger number of producers (primarily males) to obtain replacement livestock. With regard to wild boar populations, prophylactic shooting and harvesting should be carried out under the control of environmental authorities with mandatory monitoring of the state of the population gene pools.

## Conflict of interest

The authors declare no conflict of interest.
